# Investigation of electrochemical behavior of potassium ferricyanide/ferrocyanide redox probes on screen printed carbon electrode through cyclic voltammetry and electrochemical impedance spectroscopy

**DOI:** 10.3906/kim-2105-55

**Published:** 2021-08-22

**Authors:** Yücel KOÇ, Uğur MORALI, Salim EROL, Hüseyin AVCI

**Affiliations:** 1 Department of Chemical Engineering, Eskişehir Osmangazi University, Eskişehir, Turkey; 2 Department of Metallurgical and Materials Engineering, Eskişehir Osmangazi University, Eşkisehir, Turkey; 3 Cellular Therapy and Stem Cell Research Center (ESTEM), Eskişehir Osmangazi University, Eşkisehir, Turkey; 4 Translational Medicine Research and Clinical Center, Eskişehir Osmangazi University, Eskişehir, Turkey

**Keywords:** Electrochemical impedance spectroscopy, cyclic voltammetry, screen printed carbon electrode, ferricyanide-ferrocyanide redox probe, equivalent circuit modeling

## Abstract

Potassium ferricyanide, potassium ferrocyanide, and their combination system are widely used redox probes for electrochemical impedance spectroscopy (EIS) characterization. In this work, electrochemical behavior of K_3_Fe(CN)_6_, K_4_Fe(CN)_6_, and K_3_Fe(CN)_6_/K_4_Fe(CN)_6_ redox probes at five different concentrations using a screen printed carbon electrode (SPCE) by cyclic voltammetry (CV) and EIS methods was analyzed. Redox potentials were observed as a result of anodic and cathodic peak with CV analysis with determination 10 mM appropriate concentration through 0.01 mM, 0.1 mM, 1 mM, and 100 mM. In addition, with EIS analysis, each redox probe was simulated according to two different Randles circuit models and fitting equivalent model with varying concentration was determined and examined in detail. The results also demonstrated that selected high and low concentrations of redox probes can be categorized in two different models, although 1 mM behaved as a critical transition concentration. This study may contribute to the determination of relevant redox probe and its concentration in electrochemical investigations by selecting K_3_Fe(CN)_6_/K_4_Fe(CN)_6_ to decrease any risk of inaccuracy.

## 1. Introduction

In recent years, electrochemical sensors are increasingly utilized due to low cost, ease of use, portability, mass production capabilities, and simplicity of the structure. Screen printed electrodes (SPEs) are studied broadly in development of electrochemical sensors [1–3]. One of the most significant advantages of SPEs is having the ability to analyze using a small volume of analyte solution [4]. SPEs consist of a three-electrode system: working electrode, counter electrode, and reference electrode, which are generally printed using a conductive ink-based material on a solid substrate in a planar form. A trace amount of analyte sample solution can be dripped using a pipette on the electrode surface. An electrical current is generated on the analyte-SPE electrochemical system with the control of applied potential [5]. Screen-printed carbon-based electrodes (SPCEs) are an alternative material used instead of using conventional electrodes based on low background current, large potential window, high chemical stability with an economical substrate [6,7].

Electrochemical impedance spectroscopy (EIS) is a widely used technique for investigating the properties of electrode/electrolyte interface properties [8,9]. EIS is performed by measuring the alternating current resulting from applying a small sinusoidal potential perturbation. It is the ratio of potential to current, or, in other words, it is the transfer function at a certain frequency [9–11]. EIS does not alter sensor behavior during or after the measurement. Therefore, it can be identified as a noninvasive and effective tool to study sensor characteristics. EIS is a powerful method for characterizing electrochemical phenomena in sensor systems if it is carried out properly [12]. The cyclic voltammetry (CV) method can be used to study the behavior of SPEs, their potential windows, and their magnitude of background currents [13]. CV provides information on the occurrence of chemical reactions, as it is the technique commonly used to study redox reactions [14,15].

Potassium ferricyanide (K_3_Fe(CN)_6_) redox probe is the red salt composed of [Fe(CN)_6_]^3−^ coordination compound. On the other hand, potassium ferrocyanide (K_4_Fe(CN)_6_) redox probe is a yellow-green salt composed of [Fe(CN)_6_]^4−^ coordination compound. Both redox probes are water soluble and fluorescent. Potassium ferricyanide and potassium ferrocyanide are often used as a tool in physiological experiments [16–18]. K_3_Fe(CN)_6_ and K_4_Fe(CN)_6_ consist of octahedral [Fe(CN)_6_]^3−/4−^ centers cross-linked with K^+^ ions bound to CN ligands [19]. It is known that the force constant CN^−^ of [Fe(CN)_6_]^4−^ is lower than that of [Fe(CN)_6_]^3−^ [20, 21]. In the literature, among several popular reference redox systems, [Fe(CN)_6_]^3−/4−^ was chosen for its surface-sensitive electrochemical response, especially for carbon materials [14,22–24]. Information on the basic chemical properties of [Fe(CN)_6_]^3−^ and [Fe(CN)_6_]^4−^ was first reported in the 1940s [25–27]. Ribeiro et al. studied the electrical signal stability of redox probes using K_3_Fe(CN)_6_/K_4_Fe(CN)_6_ and different redox probes to monitoring the surface modification of gold-based SPE (AuSPE) [28]. Lazer et al. pointed out that when the gold electrode was used, the use of K_3_Fe(CN)_6_/K_4_Fe(CN)_6_ redox pairs would lead to the formation of polymeric complexes on the electrode surface [29]. Hocking et al. characterized multiple structures of the Fe L-edges of K_4_Fe(CN)_6_ and K_3_Fe(CN)_6_ in terms of total intensity, energy shift, and spectral shape [21].

Despite the many advantages of SPCEs, a clear representation of the electrochemical behavior of the popular redox probes (K_3_Fe(CN)_6_, K_4_Fe(CN)_6_, K_3_Fe(CN)_6_ / K_4_Fe(CN)_6_) at different concentrations has not been investigated in the literature. Therefore, using SPCE, electrochemical analysis of redox probes, which are one of the most widely used, was performed using both CV and EIS techniques. In line with this, the contribution of this work is mainly twofold. Firstly, the stability of the electrochemical signals was extensively investigated and evaluated in the context of the distinct concentrations of the most widely used redox probes such as K_3_Fe(CN)_6_, K_4_Fe(CN)_6_, and K_3_Fe(CN)_6_/K_4_Fe(CN)_6_. Secondly, the CV technique, as well as the EIS method along with the equivalent circuit modeling, were systematically implemented to determine both the redox probe and its appropriate concentration to improve electrochemical operating procedures applicable in electrochemical sensor applications.

## 2. Experimental

### 2.1. Materials

K_3_Fe(CN)_6_ and K_4_Fe(CN)_6_ were purchased from Kimetsan. Deionized (DI) water was used for preparing the solutions. Electrochemical measurements were performed with Gamry Reference 3000 Potentiostat/Galvanostat/ZRA connected to a desktop computer, controlled by Echem Analyst. Faraday cage was purchased from Gamry Instruments. SPCE (DRP-110 model) and connectors were purchased from DropSens (Spain). The working electrode, counter electrode, and reference electrode were carbon, carbon, and Ag/AgCl, respectively.

### 2.2. Methods

The volume of the redox probes at the certain concentrations used in the electrochemical measurements was approximately 50 μL. Cyclic voltammetry analysis was performed in the potential window from −0.3 to 0.5 V (vs. Ag/AgCl reference electrode). The potential scan rate was 100 mV s^−1^. Electrochemical impedance spectroscopy measurements were performed in the frequency range from 10 kHz to 0.1 Hz. The implemented potential perturbation was 1 mV vs. open circuit potential. All measurements were conducted at 22 °C. The Simplex algorithm in the Echem Analyst software was used to fit the impedance responses to the equivalent circuit model. Faraday cage was used to protect the electrochemical redox probe system from the noise and heterogeneous electric field.

## 3. Results and discussion

Electrochemical impedance spectroscopy and cyclic voltammetry measurements were performed to investigate the electrochemical behavior of the different concentrations of the redox probes on the screen-printed carbon electrode.

### 3.1. Cyclic voltammetry analysis

**Figure 1:**
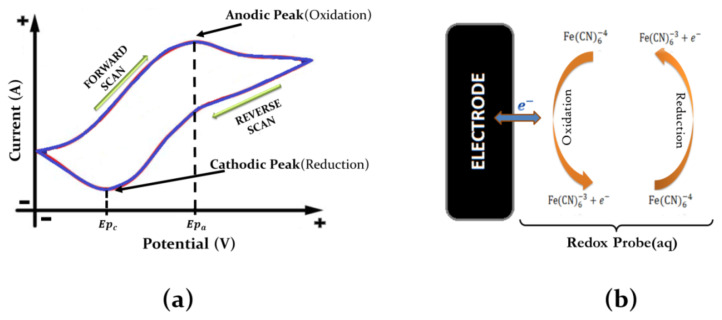
(a) The resulting cyclic voltammogram showing the measurement of the peak potentials. (b) Schematic diagram of the interface between the working electrode and the redox probe.

Cyclic voltammetry as an analytical method was used to characterize the SPCEs electrochemically. The scanning performed in the potential range gives useful information on the electrochemical properties of the working electrode of the SPCE [30]. The electrochemical behavior is presented as a voltammogram by plotting the potential range as a function of corresponding current density. A typical cyclic voltammogram is presented in Figure 1a.


(R1) $$\text{Fe}{\left(\text{CN}\right)}_{6}^{-4}\overset{\mathrm{Oxidation}}{\to }\text{Fe}{\left(\text{CN}\right)}_{6}^{-3}+e^{-}$$

(R2) $$\text{Fe}{\left(\text{CN}\right)}_{6}^{-3}+e^{-}\overset{\mathrm{Reduction}}{\to }\text{Fe}{\left(\text{CN}\right)}_{6}^{-4}$$

In this study, K_3_Fe(CN)_6_, K_4_Fe(CN)_6_, and K_3_Fe(CN)_6_/K_4_Fe(CN)_6_ were selected to determine their redox probe characteristics. The reduction/oxidation reactions occurring between the redox probe and the electrode surface are schematically presented in Figure 1b. The oxidation of [Fe(CN)_6_]^4−^ according to R1 can be observed at the anodic peak potential while the potential of the SPCE was increased from negative to positive potential. On the other hand, the reduction of [Fe(CN)_6_]^3−^ can be observed at the cathodic peak potential while the reverse potential scan was performed. The [Fe(CN)_6_]^3−^ ions are reduced to [Fe(CN)_6_]^4−^ according to R2 at the SPCE surface.

**Figure 2:**
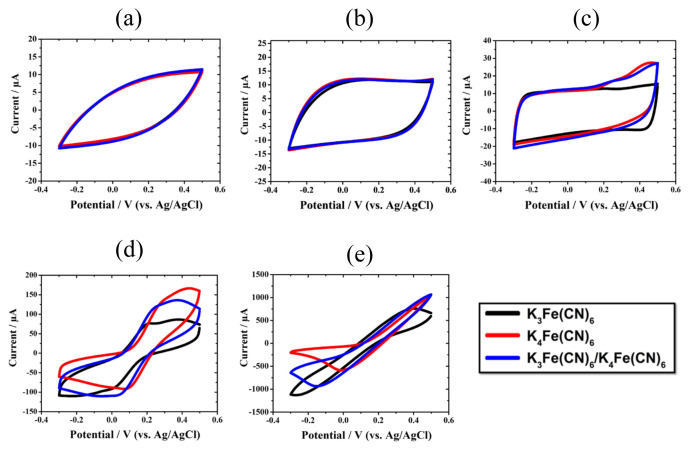
Cyclic voltammograms of SPCE recorded between −0.3 and 0.5 V potential range and 100 mV / s scan rate in the different concentrations of K_3_Fe(CN)_6_, K_4_Fe(CN)_6,_ and K_3_Fe(CN)_6_/K_4_Fe(CN)_6_; (a) 0.01 mM, (b) 0.1 mM, (c) 1 mM, (d) 10 mM, (e) 100 mM.

The cyclic voltammograms of the redox probes in the potential range of −0.3 V and 0.5 V (vs. Ag/AgCl reference electrode) are presented in Figure 2. Different concentrations of each redox probe (0.01 mM, 0.1 mM, 1 mM, 10 mM, and 100 mM) were used in the cyclic voltammetry analyses to evaluate the concentration influence on the cyclic behavior of the SPCE-redox probe system. Nearly rectangular shapes in the cyclic voltammograms presented in Figure 2a demonstrate that the electrochemical system (SPCE-redox probe) exhibited a pseudo-capacitive behavior. The increase in the concentration of each redox probe from 0.01 mM to 0.1 mM (Figure 2b) increased the current values through the potential range. This was likely due to the rectangular shape of the cyclic voltammograms in Figure 2a. The cyclic voltammograms in Figure 2c showed the rectangular shape and wide anodic peaks. The characteristic properties of redox probes, such as anodic/cathodic peak potentials and corresponding currents, are shown in Table 1. The anodic peak potentials of 0.1 mM redox probe-SPCE system were 208.9 mV for K_3_Fe(CN)_6_, 444.2 mV for K_4_Fe(CN)_6_, and 281.5 mV for K_3_Fe(CN)_6_/K_4_Fe(CN)_6_. Although the anodic peaks were observed for the electrochemical systems, the cathodic peaks in the applied potential window could not be seen, similar to the concentrations of 0.01 mM and 0.1 mM. In Figure 2d, the expected shape of the cyclic voltammograms was observed with the increase of the concentration of the redox system to 10 mM. The anodic peak currents and the potentials were 82.2 *μA*-374.3 mV for K_3_Fe(CN)_6_, 122.8 *μA*-449.1 mV for K_4_Fe(CN)_6_, and 135.3 *μA*-372.8 mV for K_3_Fe(CN)_6_/K_4_Fe(CN)_6_. In addition, the cathodic peak currents and the cathodic peak potentials were −114.10 *μA*- −230.6 mV for K_3_Fe(CN)_6_, 135.30 *μA*-69.9 mV for K_4_Fe(CN)_6_, and −111.0 *μA*- −64.5 mV for K_3_Fe(CN)_6_/K_4_Fe(CN)_6_. For the 100 mM K_3_Fe(CN)_6_, the anodic peak was observed at 401.3 mV, while the cathodic peak could not be seen in Figure 2e. On the other hand, the cathodic peak was observed for the electrochemical systems of K_4_Fe(CN)_6_ and K_3_Fe(CN)_6_/K_4_Fe(CN)_6_. However, there was no anodic peak for the K_4_Fe(CN)_6_ and K_3_Fe(CN)_6_/K_4_Fe(CN)_6_ redox probes. The results clearly showed that the concentration of the redox probe influenced the cyclic behavior. In conclusion, both anodic and cathodic peaks can only be observed using the 10 mM concentration of each redox probe in the applied potential window. Furthermore, the ratio between the anodic and cathodic peak currents (I_P,a_/I_P,c_) was only obtained at 10 mM concentration, which can be used to provide information about if the electrochemical systems were reversible.

### 3.2. Electrochemical impedance spectroscopy analysis

EIS is a powerful electroanalytical method to analyze electrochemical behaviors of electrodes. This technique along with CV method was utilized to examine the SPCEs. Solutions of K_3_Fe(CN)_6_, K_4_Fe(CN)_6_, and the combination of these two redox probes at different concentrations were used to investigate the charge transfer kinetics, mass transfer of ions, and electroanalytical performance of SPCE at the electrode/electrolyte interface.

**Figure 3:**
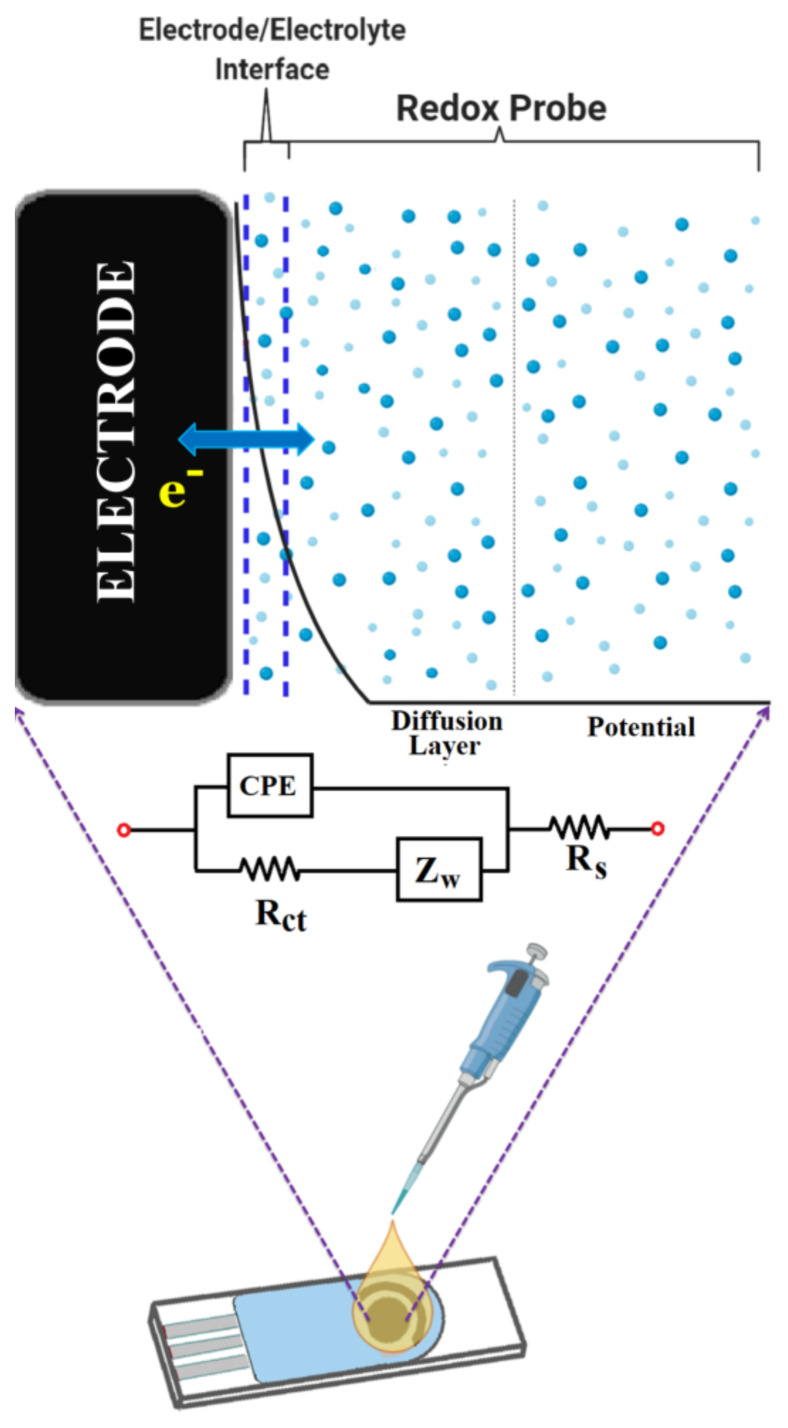
A schematic illustration for an electrode/electrolyte interface in a SPCE and corresponding equivalent Randles circuit model.

In the SPCEs, the electron transfer mechanism refers to the transition between the electrolyte and the charged ions at the electrode interface from one carrier to another. When the electrode is positively charged, negative ions in the electrolyte are attracted to the electrode/electrolyte interface. They diffuse to the interface, are absorbed onto the electrode surface, and the electrochemical reaction occurrs. This mechanism is demonstrated in Figure 3 along with the corresponding equivalent circuit.

#### 3.2.1. Equivalent circuit models

**Figure 4:**
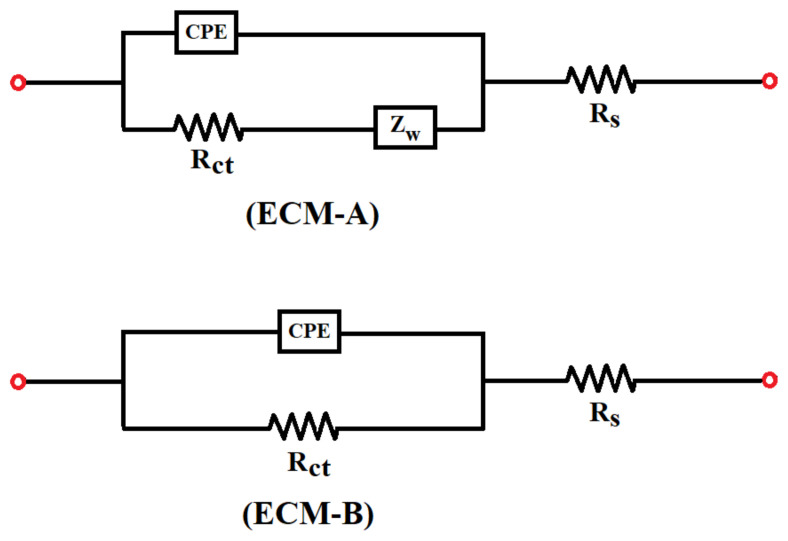
Equivalent circuit model for the SPCE electrochemical system, with Warburg element (ECM-A) and without Warburg element (ECM-B).

Impedance data of electrochemical systems are analyzed and interpreted using equivalent circuit models (ECMs). In this study, two different ECMs illustrated as in Figure 4 for the SPCE systems to analyze their impedance data.

There are four different elements in the equivalent circuits shown in Figure 4. R_s_ stands for electrolyte solution resistance, R_ct_ is charge transfer resistance, CPE is constant phase element, and Z_w_ is representing Warburg impedance. The impedance equations of the two circuit elements were calculated using the general impedance equations of these four elements in Equation (1) [31–33].


(1) $$Z_{1}=R_{\text{s}},\;\;\;Z_{2}=Z_{CPE}=\frac{1}{Q{\left(\text{j}\omega \right)}^{a}},Z_{3}=R_{\text{ct}},\;\;\;Z_{4}=Z_{\text{w}}=\frac{A_{\text{w}}}{\sqrt{\text{j}\omega }}$$

where *Q* and *α* are CPE parameters, *A*_w_ is Warburg coefficient. *Q* is called CPE coefficient, and *α* is CPE exponent.

For the equivalent circuit model A (ECM-A), the overall impedance Z_A_ is defined as


(2) $$Z_{1}=R_{s},\;\;\;Z_{2}=Z_{CPE}=\frac{1}{Q{\left(\text{j}\omega \right)}^{a}},Z_{3}=R_{\text{ct}},\;\;\;Z_{4}=Z_{\text{w}}=\frac{A_{\text{w}}}{\sqrt{\text{j}\omega }}$$

For the equivalent circuit model B (ECM-B), the overall impedance Z_B_ is defined as


(3) $$Z_{\text{B}}=Z_{1}+{\left[\frac{1}{Z_{2}}+\frac{1}{Z_{3}}\right]}^{-1}=R_{s}+\frac{R_{ct}}{1+{\left(j\omega \right)}^{\alpha }{\left(R_{ct}\right)}^{\text{Q}}}$$

In the ECM, *R*_s_ refers to the resistance of the electrolyte solution, which is an important factor in overall impedance. The resistance of the solution varies depending on the type, temperature, and concentration of the redox probe. *R*_ct_, expressed as charge transfer resistance, refers to the resistance of electrochemical reactions occurring at the electrolyte and electrode interface depending on the potential. The constant phase element, CPE, defines the capacity of electrochemical reactions that take place at the electrode/electrolyte interface and distribution of current on the electrode. Furthermore, Warburg element, *Z*_w_, expresses impedance of ion diffusion to the electrode.

#### 3.2.2. Potassium ferricyanide (K_3_Fe(CN)_6_)

**Figure 5:**
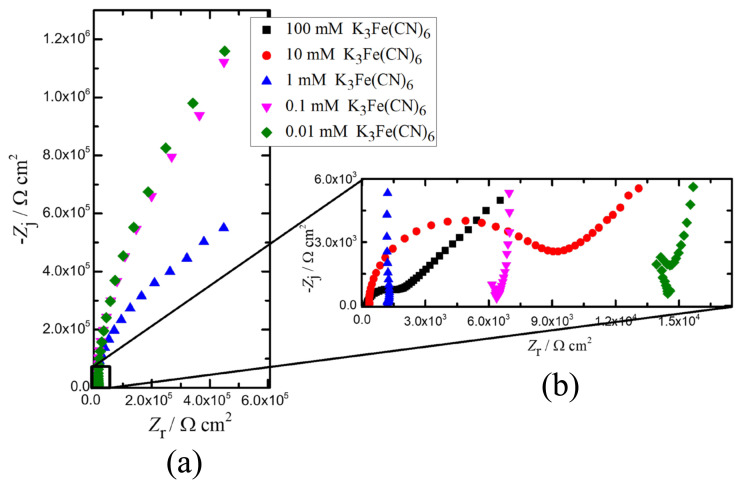
Impedance responses of different concentrations of K_3_Fe(CN)_6_ in the Nyquist format, a) complete spectra, and b) impedance response in the high frequency region.

The results obtained from K_3_Fe(CN)_6_ in the Nyquist format are presented in Figure 5a. The influence of the redox probe concentration on the impedance response at high frequency region was clearly shown in Figure 5b. Compared to the lower concentrations, the impedance responses of both 10 and 100 mM K_3_Fe(CN)_6_ exhibited semi-circle at the high frequencies called capacitive loops and low frequency lines representing ion diffusion. Controversially, the impedance responses of 0.1 and 0.01 mM K_3_Fe(CN)_6_ solutions at medium and low frequencies have similar tendencies. On the other hand, the impedance response of 1 mM K_3_Fe(CN)_6_ solution is in between the impedance responses of those higher and lower concentrated redox probes. Thus, there is a strong dependency between the concentration of K_3_Fe(CN)_6_ solution and corresponding impedance behavior. The equivalent circuit models were used to extract the physically meaningful model parameters to evaluate the electrochemical behavior of the redox probe K_3_Fe(CN)_6_-SPCE system. The regression results are presented in Table 2.

**Figure 6:**
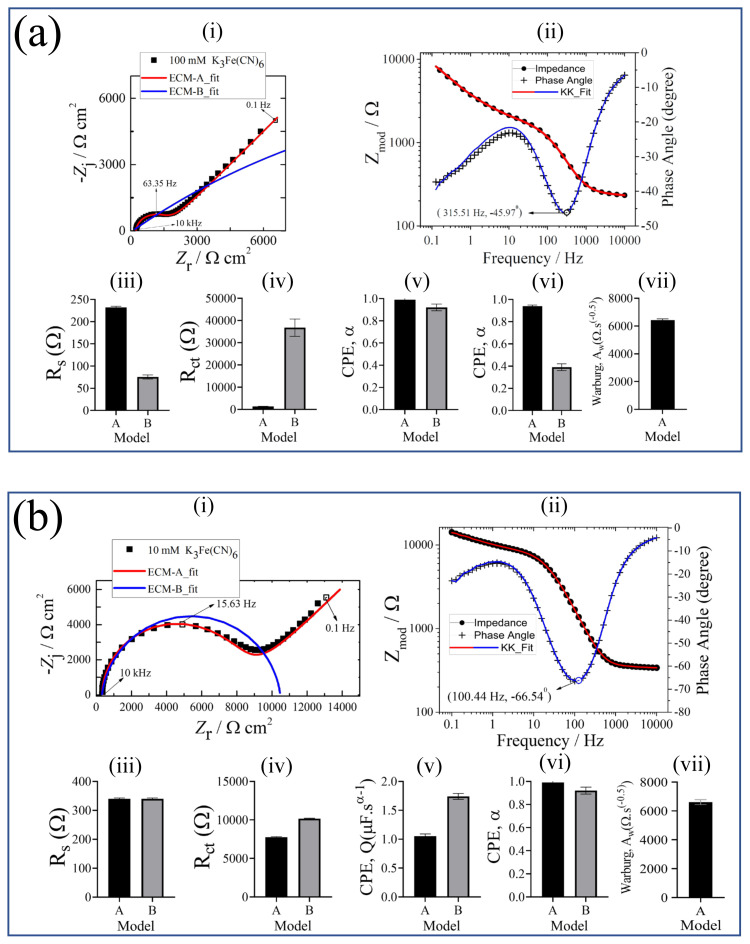

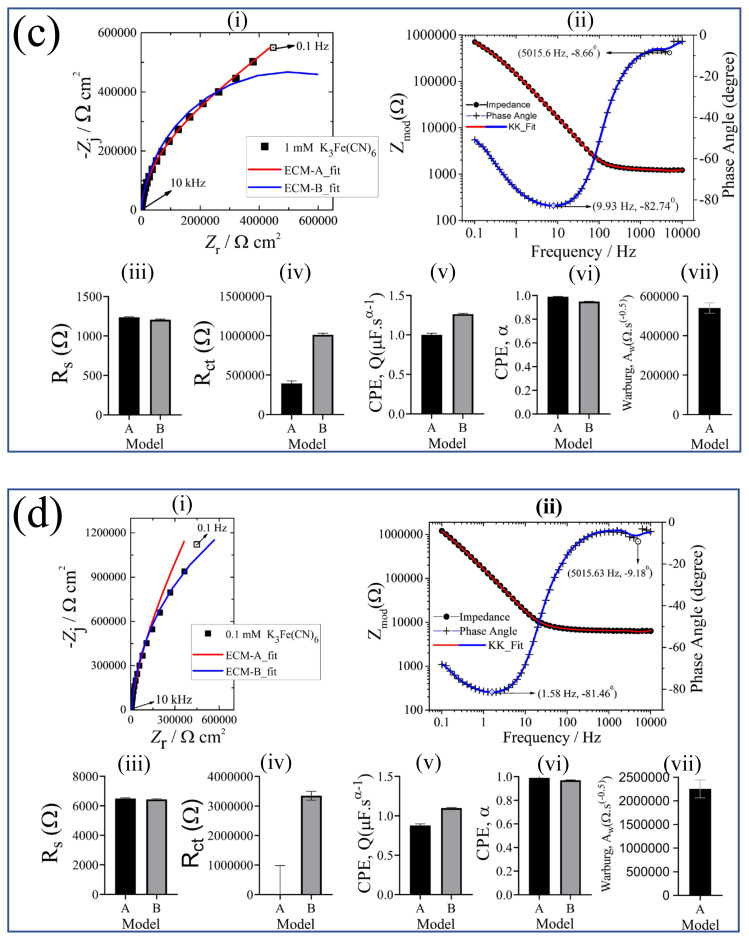

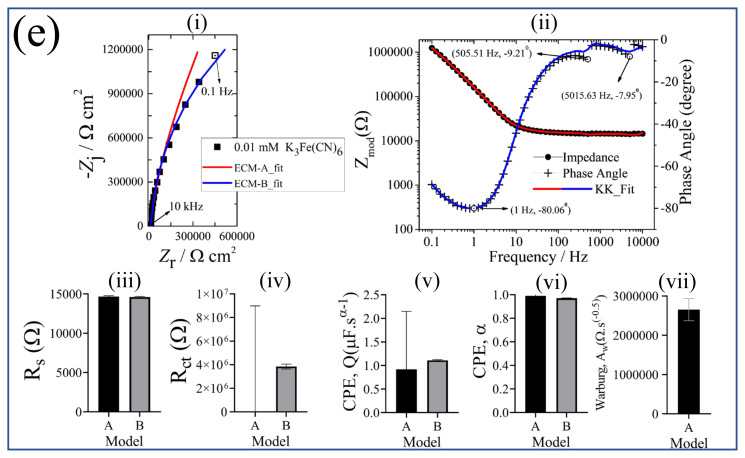
(a) 100 mM K_3_Fe(CN)_6_, (b) 10 mM K_3_Fe(CN)_6_, (c) 1 mM K_3_Fe(CN)_6_, (d) 0.1 mM K_3_Fe(CN)_6_, (e) 0.01 mM K_3_Fe(CN)_6_, (i) Nyquist plot: K_3_Fe(CN)_6_ and fitting to A and B circuit models, (ii) Bode plot of K_3_Fe(CN)_6_, Kramers–Kronig test is applied to check for the linearity and stability of the obtained data. Corresponding Nyquist plot results: (iii) Ohmic resistance, *R*_s_, represents the contact resistance from the electrode and electrolyte solution, (iv) charge-transfer resistance, *R*_ct_, represents the electrochemical reactions occurring at the electrode/electrolyte interface, (v) CPE coefficient, *Q*, shows the capacitive behavior at the electrode/electrolyte interface, (vi) CPE exponent, *α*, indicates the surface roughness and current distribution on the electrode, (vii) Warburg coefficient is related to the mass transfer phenomena of analyte.

The impedance response of 100 mM K_3_Fe(CN)_6_ is presented with the model fits in Figure 6a(i). The impedance data were validated by the Kramers–Kronig relation shown in Figure 6a(ii). The parameter values with their error bars are represented in Figure 6a(iii–vii). The results show that the values of each model parameter obtained by ECM-A were different than the ECM-B. The high error values of fitting parameters and the fit itself obtained by ECM-B imply that this model does not reflect the electrochemical behavior of the system. In addition, the CPE exponent, *α*, lower than 0.5 value obtained with ECM-B indicated that the capacitive behavior presented by ECM-B. On the contrary, the higher *α* value close to 1 by the implementation of ECM-A shows the accuracy of the model. Furthermore, the diffusion behavior of the ions represented by the straight line observed in the low frequency range can be reflected by using ECM-A only. Moreover, the value of goodness of fit for ECM-A (577.3 × 10^−6^) was lower than that of the ECM-B (50.96 × 10^−3^). In conclusion, the regressed values of the parameters, their corresponding errors, and the entire fit of the model (indicated by the goodness of fit values) indicate that ECM-A can be used to identify the impedance behavior of 100 mM K_3_Fe(CN)_6_ solution.

The Nyquist plot of 10 mM K_3_Fe(CN)_6_ in Figure 6b(i) showed a semicircle followed by a straight line. The impedance data were validated by the Kramers–Kronig relation shown in Figure 6b(ii). The parameter values with their error bars are represented in Figure 6b(iii–vii). The results showed that the value of each model parameter obtained by ECM-A was close to that of ECM-B. The error values of ECM-B were also lower than those of ECM-A. However, ECM-B did not identify the diffusion behavior of the electrochemical system that was reflected by the straight line at the low frequencies. In other words, a complete identification of the electrochemical behavior of the system was achieved by ECM-A. On the other hand, the value of goodness of fit for ECM-A (621.9 × 10^−6^) was lower than that of the ECM-B (26.06 × 10^−3^). The lower goodness of fit value indicated that the ECM-A modeled more impedance values than the ECM-B, enabling more reliable results to the model parameters. Therefore, ECM-A can be used to extract the physically meaningful parameters if the diffusion behavior is of interest.

The Nyquist plot of 10 mM K_3_Fe(CN)_6_ presented in Figure 6b(i) showed that 1 mM concentration of K_3_Fe(CN)_6_ (Figure 6c(i)) exhibited different electrochemical behavior than that of 10 mM of K_3_Fe(CN)_6_. The impedance data were validated by the Kramers–Kronig relation shown in Figure 6c(ii). The parameter values with their error bars are represented in Figure 6c(iii–vii). The fitting results indicated that ECM-A was modeled the complete impedance data, compared to ECM-B. On the other hand, the error values of the extracted model parameters of ECM-B were smaller than ECM-A. The obtained values of the ohmic resistance and the CPE exponent were similar for both ECM. Furthermore, it was observed that the sum of the charge transfer resistance and the Warburg coefficient obtained by ECM-A was close to the charge transfer resistance obtained by ECM-B. Although the goodness of fit value was close to the ECM-B, the value of goodness of fit for ECM-A (1.744 × 10^−3^) was lower than that of the ECM-B (6.917 × 10^−3^). The fitting results showed that not only the error values should be evaluated but also the physical meanings of the extracted model parameters should be taken into account. Concerning the biosensor studies, the diffusion of the ions is of great importance to get detailed information about the redox probe-sensor system. Therefore, ECM-A can be preferred to investigate such an electrochemical sensor system.

The fitting results of 0.1 mM K_3_Fe(CN)_6_ presented in Figure 6d(i) showed that the error values from ECM-A were higher than that of ECM-B. The impedance data were validated by the Kramers–Kronig relation shown in Figure 6d(ii). The parameter values with their error bars are represented in Figure 6d(iii–vii). This result was also supported by the goodness of fit values in Table 3. The extracted model parameters showed that the sum of the values of the charge transfer resistance and the coefficient of Warburg impedance from ECM-A was close to the charge transfer resistance from ECM-B. This behavior was also similar to that observed at 1 mM K_3_Fe(CN)_6_. It could be attributed to the low concentration of K_3_Fe(CN)_6_. The results indicated that the higher concentration of K_3_Fe(CN)_6_ can be used to separate the capacitive behavior and diffusion behavior of the system.

Similar fitting results to 0.1 mM K_3_Fe(CN)_6_ were obtained on the Nyquist plot of 0.01 mM K_3_Fe(CN)_6_ (Figure 6e(i)). The impedance data were validated by the Kramers–Kronig relation shown in Figure 6e(ii). The parameter values with their error bars are represented in Figure 6e(iii–vii). Similar to the goodness of fit values at 0.1 mM concentration, the goodness of fit value of the ECM-A was higher than the ECM-B. The charge transfer resistance obtained from ECM-A exhibited a higher error value, compared to that at 0.1 mM K_3_Fe(CN)_6_. This could also be attributed to the low concentration of K_3_Fe(CN)_6_. Furthermore, the error of the coefficient of Warburg impedance at 0.01 mM of K_3_Fe(CN)_6_ was higher than that at 0.1 mM K_3_Fe(CN)_6_. This was also probably due to the low concentration of K_3_Fe(CN)_6_ solution. This result showed that the K_3_Fe(CN)_6_ only interacted with the surface of the SPCE. In addition, this result indicated that the low concentration of K_3_Fe(CN)_6_ restricted the diffusion of the ions to the electrode. It is important to emphasize that higher concentration of K_3_Fe(CN)_6_ than 100 mM will enable to electrochemically investigate both capacitive behavior and the diffusion mechanism of the ions in the frequency range implemented in this work.

#### 3.2.3. Potassium ferrocyanide (K_4_Fe(CN)_6_)

**Figure 7:**
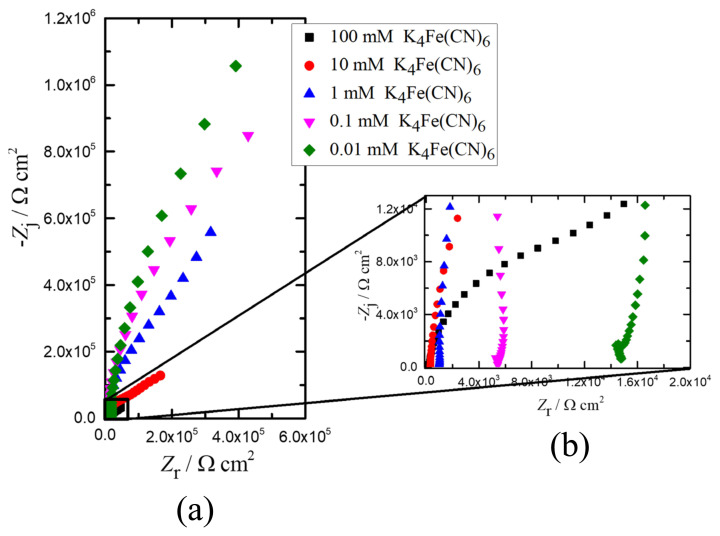
Impedance responses of different concentrations of K_4_Fe(CN)_6_ in the Nyquist format, a) complete spectra and b) impedance response in the high frequency region.

**Figure 8:**
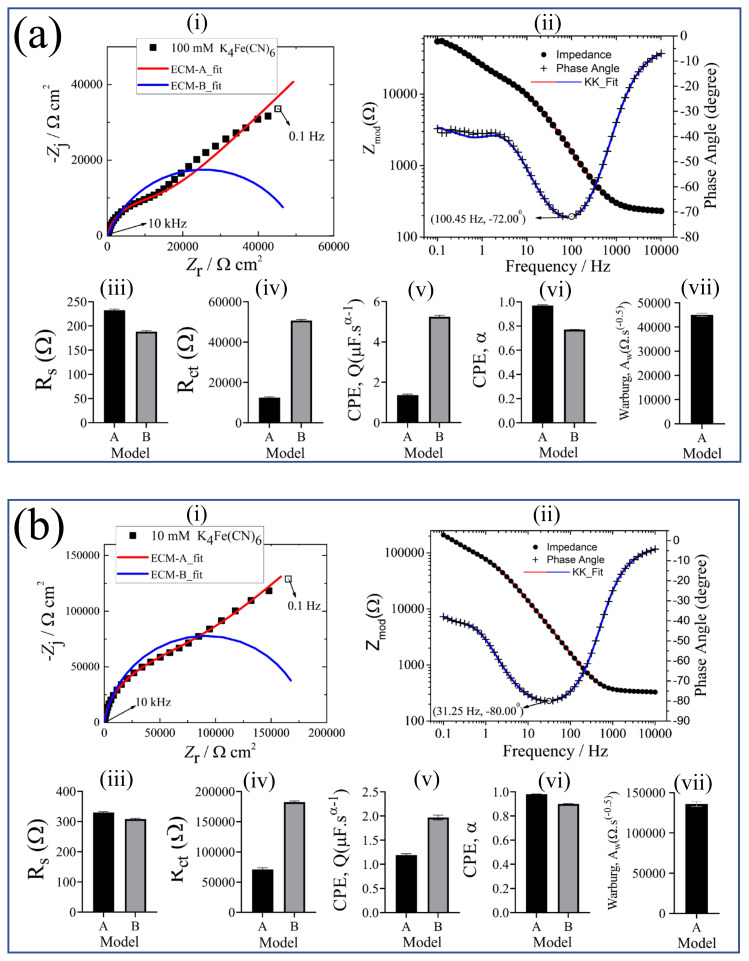

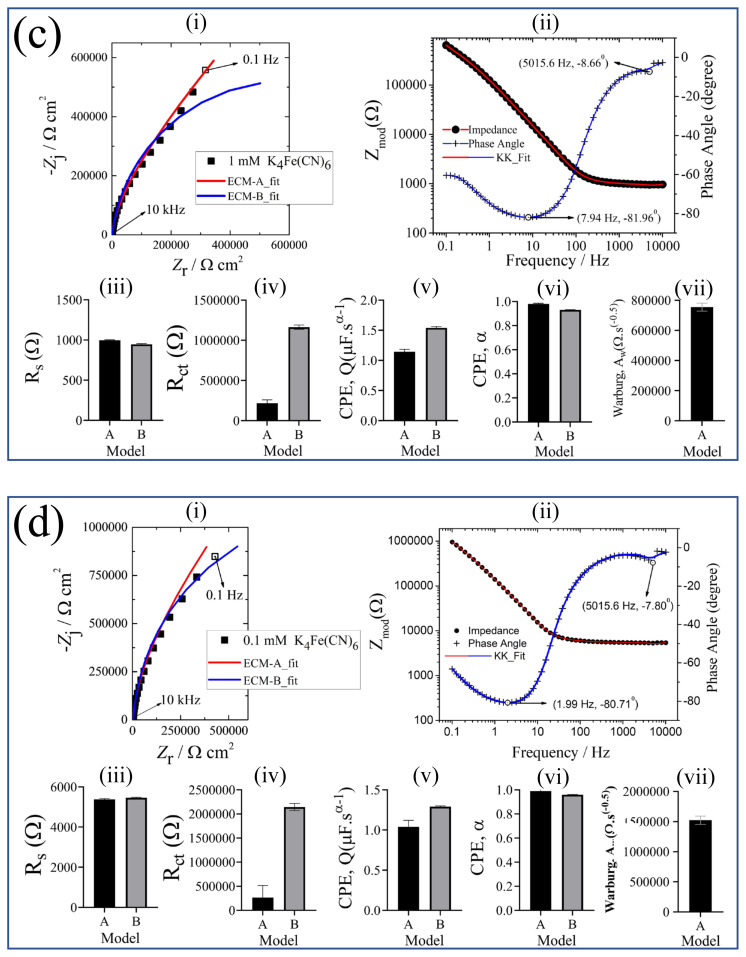

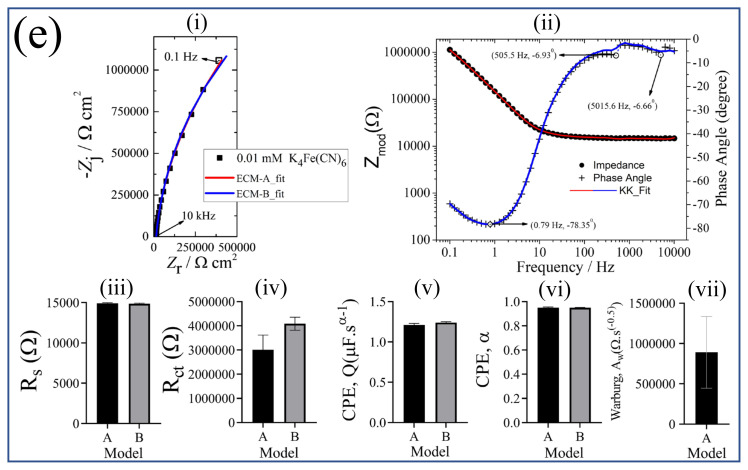
(a) 100 mM K_4_Fe(CN)_6_, (b) 10 mM K_4_Fe(CN)_6_, (c) 1 mM K_4_Fe(CN)_6_, (d) 0.1 mM K_4_Fe(CN)_6_, (e) 0.01 mM K_4_Fe(CN)_6_; (i) Nyquist plot: K_4_Fe(CN)_6_ and fitting to A and B circuit models, (ii) Bode plot of K_4_Fe(CN)_6_, Kramers-Kronig test is applied to check for the linearity and stability of the obtained data. Corresponding Nyquist plot results; (iii) Ohmic resistance, *R*_s_, represents the contact resistance from the electrode and electrolyte solution, (iv) charge-transfer resistance, *R*_ct_, represents the electrochemical reactions occurring at the electrode/electrolyte interface, (v) CPE coefficient, *Q*, shows the capacitive behavior at the electrode/electrolyte interface, (vi) CPE exponent, *α*, indicates the surface roughness and current distribution on the electrode, (vii) Warburg coefficient is related to the mass transfer phenomena of analyte.

The impedance response of K_4_Fe(CN)_6_ in the Nyquist format are presented in Figure 7a. Figure 7b shows the high frequency region impedance response. The regressed model parameters obtained via Nyquist graph as a result of fitting K_4_Fe(CN)_6_ in different concentrations according to two different models with Bode graph by validating the Kramers-Kronig relation are shown in Figure 8. In Figure 8a(i), 8b(i), and 8c(i), the Nyquist plot of 100 mM, 10 mM, and 1 mM for K_4_Fe(CN)_6_, as in K_3_Fe(CN)_6_, first semicircle and then linear diffusion according to the error bars in the impedance results, ECM-A was resulted as an appropriate model. This result was also supported by the lower goodness of fit value of the ECM-A than the ECM-B. When the Nyquist plots of 0.1 mM and 0.01 mM K_4_Fe(CN)_6_ were examined in Figure 8d(i) and 8e(i), it is realized that there was no full semicircle and linear diffusion as in K_3_Fe(CN)_6_, and, according to the error bars in the impedance results, ECM-B was found to be the appropriate model. The impedance data were validated by the Kramers–Kronig relation shown in Figure 8a–8e(ii). The parameter values with their error bars are represented in Figure 8a–8e(iii–vii). These results were supported by the regressed model parameters in Table 4 and the goodness of fit values in Table 3. In addition, after the Bode plots were investigated, Kramers–Kronig relation was seen, and the distortions in the starting frequencies at low concentrations (1 mM, 0.1 mM, 0.01 mM) repeat as in K_3_Fe(CN)_6_.

#### 3.2.4. Potassium ferricyanide/ferrocyanide (K_3_Fe(CN)_6_/K_4_Fe(CN)_6_)

**Figure 9:**
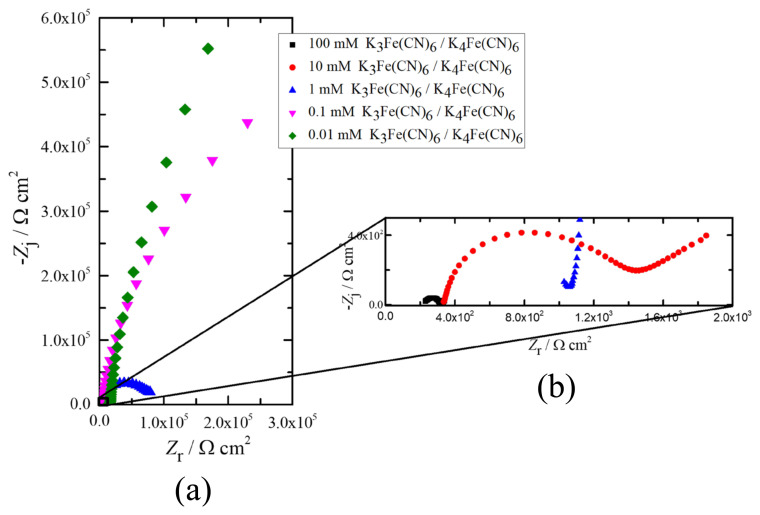
Impedance responses of different concentrations of K_3_Fe(CN)_6_/K_4_Fe(CN)_6_ in the Nyquist format. a) Complete spectra and b) impedance response in the high frequency region.

**Figure 10:**
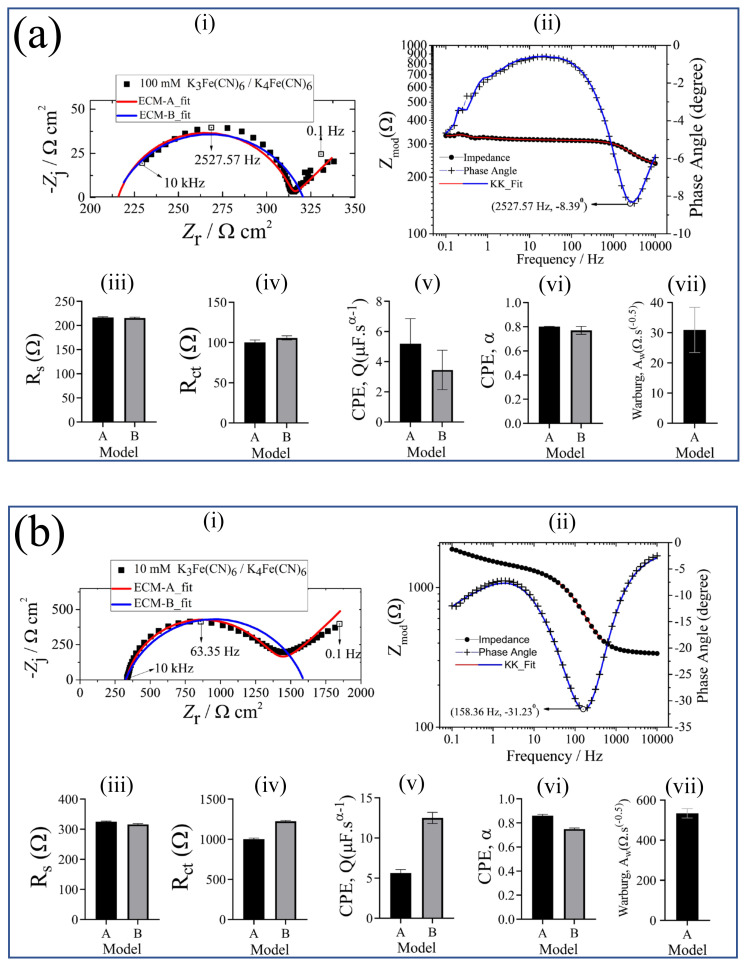

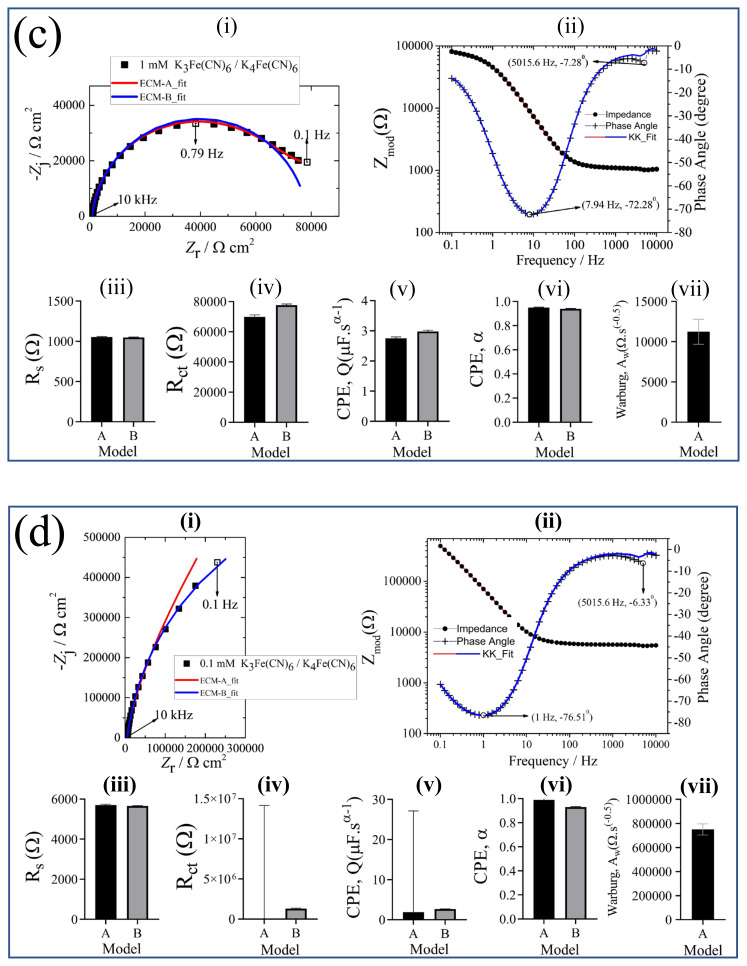

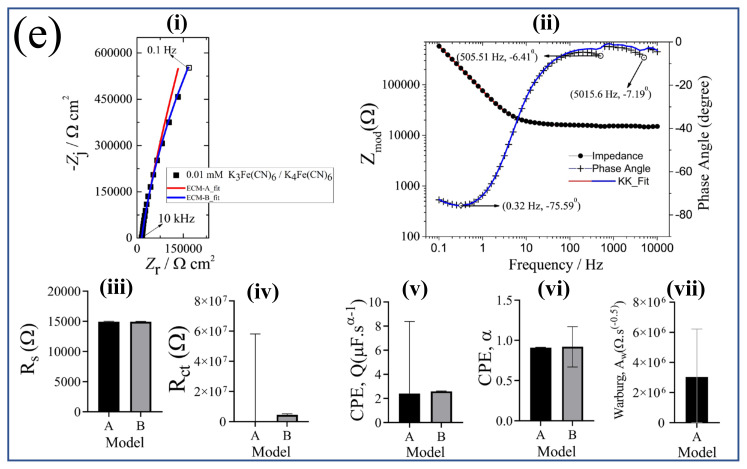
Data obtained by using (a) 100 mM K_3_Fe(CN)_6_/K_4_Fe(CN)_6_, (b) 10 mM K_3_Fe(CN)_6_/K_4_Fe(CN)_6_, (c) 1 mM K_3_Fe(CN)_6_/K_4_Fe(CN)_6_, (d) 0.1 mM K_3_Fe(CN)_6_/K_4_Fe(CN)_6_, (e) 0.01 mM K_3_Fe(CN)_6_/K_4_Fe(CN)_6_; (i) Nyquist plot: K_3_Fe(CN)_6_/K_4_Fe(CN)_6_ and fitting to A and B circuit models, (ii) Bode plot of K_4_Fe(CN)_6_, Kramers–Kronig relations test was applied to check for the linearity and stability of the data. Corresponding Nyquist plot results; (iii) Ohmic resistance, *R*_s_, represents the contact resistance from the electrode and electrolyte solution, (iv) charge-transfer resistance, *R*_ct_, shows the electrochemical reactions occurring at the electrode/electrolyte interface, (v) CPE coefficient, *Q*, demonstrates the capacitive behavior at the electrode/electrolyte interface, (vi) CPE exponent, *α*, indicates the surface roughness and current distribution on the electrode, (vii) Warburg coefficient is related to the mass transfer phenomena of the analyte.

The impedance response of K_3_Fe(CN)_6_/K_4_Fe(CN)_6_ in the Nyquist format are presented in Figure 9a(i). Figure 9b shows the impedance response at high frequencies. As seen in Figure 10a–10e(i), Nyquist graph has been obtained as a result of fitting K_3_Fe(CN)_6_/K_4_Fe(CN)_6_ at different concentrations according to two different models, and a Bode plot of Kramers–Kronig relation was tested, as shown in Figure 10a–10e(ii). The semicircle of Nyquist plots by using K_3_Fe(CN)_6_/K_4_Fe(CN)_6_ of 100 mM, 10 mM, and 1 mM, respectively, were more like a full half circle compared to the ones were obtained via K_3_Fe(CN)_6_ ve K_4_Fe(CN)_6_ (Figure 10a(i), 10b(i) and 10c(i)). When the 100 mM Nyquist and Bode plot were examined in Figure 10a(i) and Figure 10a(ii), respectively, it has been observed that there were distortions in the low frequencies. It can be concluded that ECM-A was more appropriate when the impedance results were examined according to the error bars. Furthermore, the goodness of fit value of the ECM-A and ECM-B was 154.5 × 10^−6^ and 392.2 × 10^−6^, respectively. The lower fit value also indicated that the ECM-A was more suitable to modeling the impedance responses of 100 mM K_3_Fe(CN)_6_/K_4_Fe(CN)_6_. The goodness of fit value of ECM-A (554.6 × 10^−6^) was considerably lower than that of the ECM-B (7.752 × 10^−3^). In addition, after investigation of the 10 mM K_3_Fe(CN)_6_/K_4_Fe(CN)_6_ Nyquist and Bode plot in Figure 10b(i) and Figure 10b(ii), respectively, it was seen that Kramers–Kronig relations and ECM-A were more suitable according to the impedance results. The Nyquist graph by using 1 mM K_3_Fe(CN)_6_/K_4_Fe(CN)_6_ only formed a half circle with the lack of linear diffusion (Figure 10c(i)). When the Bode plot in Figure 10c(ii) was examined, the Kramers–Kronig relations was seen but as in other solutions, there were distortions from the initial frequency of 10000 Hz to 5015.6 Hz. The ECM-A was more suitable model according to the impedance results. This was also supported by the lower goodness of fit value of the ECM-A (1.647 × 10^−3^) in Table 3. Based on the Nyquist graphs of 0.1 mM and 0.01 mM K_3_Fe(CN)_6_/K_4_Fe(CN)_6_ in Figure 10d(i) and Figure 10e(i), as in K_3_Fe(CN)_6_ and K_4_Fe(CN)_6_, a full semicircle and linear diffusion did not occur, and ECM-B was looking more appropriate model according to the error bars in the impedance results and the lower goodness of fit values. The regressed model parameters (Figure 10 c–10e (iii–vii)) and corresponding error values shown in Table 5 also supported these results. When the Bode plots were examined, the Kramers–Kronig relations have been obtained, and the distortions were again repeated in the starting frequencies.

### 3.3. Comparison of equivalent circuit model parameters of each redox probe

**Figure 11:**
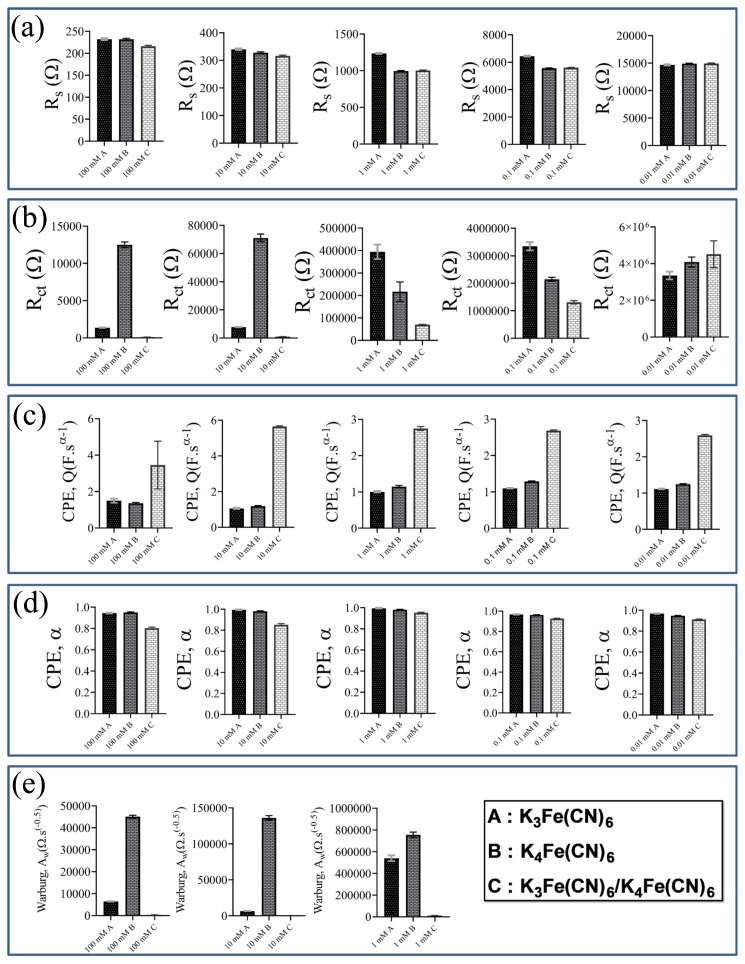
Equivalent circuit model parameters obtained by using the most convenient model for each redox probe at various concentrations; (a) ohmic resistance R_s_, (b) charge transfer resistance *R*_ct_, (c) CPE coefficient *Q*, (d) CPE exponent *α*, (e) coefficient of Warburg impedance.

The ECM-A was used to extract the physically meaningful parameters from the impedance responses obtained at concentrations from 1 to 100 mM of each redox probe. The ECM-B was implemented to obtain the components of the equivalent circuit model fitted to the impedance responses obtained at 0.1 and 0.01 mM of each redox probe. The equivalent circuit model parameters of each redox probe at various concentrations are presented in Figure 11 to clearly observe the influence of both redox probe and concentration on the parameters.

The ohmic resistance of each redox probe at various concentrations (100, 10, 1, 0.1, 0.01 mM) is presented in Figure 11 (a). The ohmic resistance was decreased with the increasing concentration of each redox probe. In other words, the highest and the lowest ohmic resistances were obtained at the concentration of 0.01 mM and 100 mM of each redox probe, respectively. The ohmic resistance of K_3_Fe(CN)_6_ was the highest one at each concentration, except at 0.01 mM concentration. The lowest ohmic resistance at 100 mM was obtained for the K_3_Fe(CN)_6_/K_4_Fe(CN)_6_ solution, likely due to synergistic influence of redox probe. The ohmic resistance of K_4_Fe(CN)_6_ at 100 mM concentration was similar to that of K_3_Fe(CN)_6_. Furthermore, the highest difference between the ohmic resistance values was observed at the moderate concentration of 1 mM. On the other hand, the ohmic resistance of K_4_Fe(CN)_6_ was closer to that of K_3_Fe(CN)_6_/K_4_Fe(CN)_6_ as the concentration decreased from 1 to 0.01 mM. Moreover, the ohmic resistance values of each redox probe at 0.01 mM concentration were similar to each other.

The charge transfer resistance of each redox probe at different concentrations is displayed in Figure 11 (b). The charge transfer resistance of each redox probe was increased by decreasing the concentration from 100 to 0.01 mM. The highest charge transfer resistance was obtained by using 0.01 mM K_3_Fe(CN)_6_/K_4_Fe(CN)_6_ redox probe. Furthermore, the lowest charge transfer resistance was calculated for the K_3_Fe(CN)_6_/K_4_Fe(CN)_6_ solution when its concentration was 100 mM. The highest charge transfer resistance at 100 and 10 mM concentration was observed for the K_4_Fe(CN)_6_ redox probe. On the other hand, the K_3_Fe(CN)_6_ redox probe at 1 and 0.1 mM concentrations exhibited the highest charge transfer resistance. These results demonstrated that the concentration of the redox probe solutions considerably influenced the charge transfer resistance.

The CPE coefficient of each redox probe is shown in Figure 11 (c). The CPE coefficient indicates the capacitive behavior of the system. The K_3_Fe(CN)_6_/K_4_Fe(CN)_6_ redox probe at each concentration exhibited the highest CPE coefficient value. This could be attributed to the K_3_Fe(CN)_6_/K_4_Fe(CN)_6_ behavior on the screen printed electrode. However, the CPE coefficient of K_4_Fe(CN)_6_ redox probe was higher than the K_3_Fe(CN)_6_ redox probe, except at 100 mM concentration.

The CPE exponent α indicates the homogeneity of the current density on the screen-printed electrode. The value of the CPE exponent should be in the range of 0 < α < 1. The values of α are shown in Figure 11 (d). The CPE exponent decreased with decreasing the concentration of redox probes. All the values of the CPE exponent higher than 0.8 indicated the homogenous current distribution on the surface of the screen-printed electrode. Furthermore, the value of the CPE exponent indicates the surface roughness of the electrode. The high values of α for each redox probe indicated the smooth surface of the electrodes. The highest α value was observed for K_3_Fe(CN)_6_ at 1 mM concentration. This showed that the more homogenous current density could be obtained by using K_3_Fe(CN)_6_ redox probe at 1 mM.

The Warburg coefficients are presented in Figure 11 (e). The Warburg coefficient was increased with decreasing the concentration from 100 to 1 mM. The highest Warburg coefficient was obtained for the K_4_Fe(CN)_6_. Compared to the K_4_Fe(CN)_6_ and the K_3_Fe(CN)_6_/K_4_Fe(CN)_6_, the redox probe K_3_Fe(CN)_6_ exhibited the moderate Warburg coefficient value. On the other hand, it was clearly observed that the Warburg coefficient of the K_3_Fe(CN)_6_/K_4_Fe(CN)_6_ was clearly lower than those of the K_3_Fe(CN)_6_ and the K_4_Fe(CN)_6_. Furthermore, it was important to note that the trend for the Warburg coefficient versus concentration was similar to the observed for the charge transfer coefficient.

## 4. Conclusion

In this study, electrochemical analysis of three different redox probes of K_3_Fe(CN)_6_, K_4_Fe(CN)_6_, and K_3_Fe(CN)_6_/K_4_Fe(CN)_6_ at five concentrations was performed using two different electrochemical analysis techniques of cyclic voltammetry and electrochemical impedance spectroscopy. Anodic and cathodic peak analysis of redox probes were investigated with CV analysis. It was determined by CV analysis that the redox probe at a concentration of 10 mM gave both anodic and cathodic peak from three different redox probes at 0.01 mM, 0.1 mM, 1 mM, 10 mM, and 100 mM concentrations. With this result, it has been shown that it is necessary to determine the optimum concentration in studies using the CV technique.

With EIS analysis, the raw data of redox probes were simulated and then evaluated using two different Randles circuit models, and the equivalent circuit model that changes with different concentration was determined and shown. It was realized that redox probes at 100 mM, 10 mM, and 1 mM concentrations can be modeled with ECM-A containing Warburg diffusion element, and redox probes with a concentration of 0.1 mM and 0.01 mM indicate with ECM-B without Warburg diffusion element.

The detailed findings reported in this work recommend to find starting point of an appropriate and optimum redox probe from widely used ones for EIS characterization of chemically modified electrodes. Depending on applied potential and the structure of chemical modification on the electrode, ferricyanide ions can be adsorbed or diffused to the layer; therefore, K_3_Fe(CN)_6_/K_4_Fe(CN)_6_ might be preferred to eliminate any risk of inaccuracy.

## Figures and Tables

**Table 1:** Characteristic properties of redox probes obtained from Figure 2.

Redox Probe	I_P(a)_, μA	I_P(c)_, μA	E_P(a)_, mV	E_P(c)_, mV	n = I_P(a)_ / I_P(c)_
100 mM K_3_Fe(CN)_6_	758.5	None	401.3	None	-
10 mM K_3_Fe(CN)_6_	82.2	−114.10	374.3	−230.6	0.72
1 mM K_3_Fe(CN)_6_	12.67	None	208.9	None	-
0.1 mM K_3_Fe(CN)_6_	11.83	None	167.9	None	-
0.01 mM K_3_Fe(CN)_6_	None	None	None	None	-
100 mM K_4_Fe(CN)_6_	None	− 583.60	None	− 9.1	-
10 mM K_4_Fe(CN)_6_	122.80	135.30	449.1	69.9	0.90
1 mM K_4_Fe(CN)_6_	28.34	None	444.2	None	-
0.1 mM K_4_Fe(CN)_6_	12.24	None	100.8	None	-
0.01 mM K_4_Fe(CN)_6_	None	None	None	None	-
100 mM K_3_Fe(CN)_6_/K_4_Fe(CN)_6_	None	− 985.50	None	− 156.8	-
10 mM K_3_Fe(CN)_6_/K_4_Fe(CN)_6_	135.3	− 111.00	372.8	− 64.5	1.21
1 mM K_3_Fe(CN)_6_/K_4_Fe(CN)_6_	20.45	None	281.5	None	-
0.1 mM K_3_Fe(CN)_6_/K_4_Fe(CN)_6_	11.94	None	106.1	None	-
0.01 mM K_3_Fe(CN)_6_/K_4_Fe(CN)_6_	None	None	None	None	-

**Table 2:** Regression results and their ±σ confidence intervals for the impedance data represented in Figure 5.

Concentration of K_3_Fe(CN)_6_, mM	Model	*R*_s_, Ω	*R*_ct_, Ω	*Q*, μF s^α − 1^	*α*	*A*_w_, Ω s ^− 0.5^
100	A	232 ± 2.68	1362 ± 27.35	1.50 ± 0.12	0.94 ± 0.01	6439.15 ± 76.29
	B	75.42 ± 4.62	36770 ± 3876	109.2 ± 2.17	0.39 ± 0.003	-
10	A	339.7 ± 3.07	7740 ± 90.43	1.05 ± 0.04	0.99 ± 0.005	6618.13 ± 172.61
	B	339.7 ± 3.07	10150 ± 70.93	1.74 ± 0.05	0.92 ± 0.003	-
1	A	1235 ± 8.98	393900 ± 32120	1.002 ± 0.02014	0.99 ± 0.004	539956.80 ± 26484.71
	B	1206 ± 8.09	1011000 ± 18910	1.263 ± 0.01137	0.95 ± 0.002	-
0.1	A	6500 ± 46.09	21000 ± 961800	0.8771 ± 0.2104	0.99 ± 0.013	2252759.631 ± 189142.49
	B	6444 ± 36.45	3348000 ± 150300	1.098 ± 0.009589	0.96 ± 0.003	-
0.01	A	14670 ± 111.9	4949 ± 8983000	0.9193 ± 1.296	0.99 ± 0.014	2653223.67 ± 282569.38
	B	14600 ± 77.15	3846000 ± 207900	1.112 ± 0.01009	0.97 ± 0.003	-

**Table 3:** Goodness of fit values of each model at various concentrations of redox probes.

Concentration	Model	K_3_Fe(CN)_6_/K_4_Fe(CN)_6_	K_4_Fe(CN)_6_	K_3_Fe(CN)_6_
100	A	0.0001545	0.0015830	0.0005773
	B	0.0003922	0.0550100	0.0509600
10	A	0.0005546	0.0002301	0.0006219
	B	0.0077520	0.0247900	0.0260600
1	A	0.0016470	0.0025080	0.0017440
	B	0.0024150	0.0088780	0.0069170
0.1	A	0.0056630	0.0034880	0.0033730
	B	0.0047310	0.0034480	0.0032430
0.01	A	0.0159000	0.0024110	0.0045180
	B	0.0156100	0.0024830	0.0035370

**Table 4:** Regression results and their ±σ confidence intervals for the impedance data represented in Figure 8.

Concentration of K_4_Fe(CN)_6_, mM	Model	*R*_s_, Ω	*R*_ct_, Ω	*Q*, μF s^α − 1^	*α*	*A*_w_, Ω s ^− 0.5^
100	A	232.5 ± 2.33	12490 ± 387.3	1.36 ± 0.05	0.97 ± 0.005	45065.34 ± 660.85
	B	188.2 ± 2.21	50660 ± 568.3	5.24 ± 0.08	0.77 ± 0.002	-
10	A	329.8 ± 2.91	71060 ± 2766	1.19 ± 0.03	0.98 ± 0.003	136054.42 ± 3315.29
	B	308.2 ± 2.65	182400 ± 2060	1.97 ± 0.05	0.90 ± 0.002	-
1	A	996.1 ± 7.50	216600 ± 43200	1.144 ± 0.04	0.98 ± 0.005	754716.98 ± 26514.77
	B	947.3 ± 6.60	1164000 ± 27120	1.54 ± 0.02	0.93 ± 0.002	-
0.1	A	5378 ± 37.22	265900 ± 249200	1.04 ± 0.08	0.99 ± 0.014	1521838.38 ± 69502.92
	B	5459 ± 30.89	2144000 ± 74050	1.29 ± 0.01	0.96 ± 0.003	-
0.01	A	14900 ± 87.89	3011000 ± 607500	1.21 ± 0.02	0.95 ± 0.006	890471.95 ± 444601.62
	B	14870 ± 78.81	4089000 ± 268300	1.24 ± 0.01	0.95 ± 0.003	-

**Table 5:** Regression results and their ±σ confidence intervals for the impedance data represented in Figure 10.

Concentration of K_3_Fe(CN)_6_/ K_4_Fe(CN)_6_, mM	Model	*R*_s_, Ω	*R*_ct_, Ω	*Q*, μF s^α − 1^	*α*	*A*_w_, Ω s ^− 0.5^
100	A	216.3 ± 1.78	100.2 ± 2.98	5.19 ± 1.66	0.80 ± 0.004	30.92 ± 7.50
	B	215.5 ± 1.68	105.7 ± 2.56	3.45 ± 1.31	0.77 ± 0.033	-
10	A	325 ± 2.33	1002 ± 13.34	5.64 ± 0.43	0.86 ± 0.011	534.19 ± 23.51
	B	316.2 ± 2.25	1225 ± 11.18	12.51 ± 0.7	0.75 ± 0.008	-
1	A	1053 ± 5.93	69820 ± 1361	2.75 ± 0.05	0.95 ± 0.004	11243.53 ± 1558.72
	B	1048 ± 5.416	77640 ± 802.5	2.98 ± 0.04	0.94 ± 0.003	-
0.1	A	5702 ± 38.39	130.2 ± 14170000	1.92 ± 25.22	0.99 ± 0.120	750187.55 ± 46401.32
	B	5652 ± 26.28	1310000 ± 61750	2.68 ± 0.03	0.93 ± 0.003	-
0.01	A	14930 ± 78.08	3583 ± 58160000	2.40 ± 5.98	0.91 ± 0.004	3034901.37 ± 3178587.14
	B	14920 ± 66.92	4510000 ± 732000	2.59 ± 0.03	0.92 ± 0.251	-
